# Crohn’s Disease-Associated Adherent-Invasive *Escherichia coli* Manipulate Host Autophagy by Impairing SUMOylation

**DOI:** 10.3390/cells8010035

**Published:** 2019-01-09

**Authors:** Guillaume Dalmasso, Hang T. T. Nguyen, Tiphanie Faïs, Sébastien Massier, Nicolas Barnich, Julien Delmas, Richard Bonnet

**Affiliations:** 1 UMR 1071 Inserm, University of Clermont Auvergne, 28 place Henri Dunant, Clermont-Ferrand 63000, France; hang.nguyen@uca.fr (H.T.T.N.); tiphanie.fais@uca.fr (T.F.); seb.massier@free.fr (S.M.); nicolas.barnich@uca.fr (N.B.); jdelmas@chu-clermontferrand.fr (J.D.); 2 INRA, USC 2018, University of Clermont Auvergne, 28 place Henri Dunant, Clermont-Ferrand 63000, France; 3 Centre Hospitalier Universitaire, 58 place Montalembert, Clermont-Ferrand 63000, France

**Keywords:** Crohn’s disease, AIEC, post-translational modifications, SUMO, autophagy

## Abstract

The intestinal mucosa of Crohn’s disease (CD) patients is abnormally colonized with adherent-invasive *Escherichia coli* (AIEC) that are able to adhere to and to invade intestinal epithelial cells (IECs), to survive in macrophages, and to induce a pro-inflammatory response. AIEC persist in the intestine, and induce inflammation in CEABAC10 transgenic mice expressing human CAECAM6, the receptor for AIEC. SUMOylation is a eukaryotic-reversible post-translational modification, in which SUMO, an ubiquitin-like polypeptide, is covalently linked to target proteins. Here, we investigated the role of SUMOylation in host responses to AIEC infection. We found that infection with the AIEC LF82 reference strain markedly decreased the levels of SUMO-conjugated proteins in human intestinal epithelial T84 cells. This was also observed in IECs from LF82-infected CEABAC10 transgenic mice. LF82-induced deSUMOylation in IECs was due in part to increased level of microRNA (miR)-18, which targets *PIAS3* mRNA encoding a protein involved in SUMOylation. Over-expression of SUMOs in T84 cells induced autophagy, leading to a significant decrease in the number of intracellular LF82. Consistently, a decreased expression of UBC9, a protein necessary for SUMOylation, was accompanied with a decrease of LF82-induced autophagy, increasing bacterial intracellular proliferation and inflammation. Finally, the inhibition of miR-18 significantly decreased the number of intracellular LF82. In conclusion, our results suggest that AIEC inhibits the autophagy response to replicate intracellularly by manipulating host SUMOylation.

## 1. Introduction

Inflammatory bowel diseases (IBD), which include ulcerative colitis and Crohn’s disease (CD), are chronic gastrointestinal disorders. Extensive studies in the last few decades have shown that the etiology of IBD involves environmental and genetic factors that lead to the dysfunction of the epithelial barrier, with consequent deregulation of the mucosal immune response to gut microbiota [1]. An imbalance in gut microbiota has been observed in IBD patients with an increase in the abundance of putative pathogenic bacterial species such as *Escherichia coli* and *Bacteroides*, and a decrease of putative beneficial bacteria such as *Bifidobacteria*, *Lactobacilli*, and *Firmicutes* [2,3]. Our group and others have found a high prevalence of a pathovar of *E. coli* called AIEC for adherent-invasive *E. coli* in the ileal mucosa of CD patients [4,5,6]. AIEC have been shown to adhere to and to invade intestinal epithelial cells (IECs), to survive and replicate inside macrophages without inducing cell death, and to induce a high production of pro-inflammatory cytokines and chemiokines [2,3]. AIEC adhere to enterocytes via the interaction between type 1 pili and the host receptor carcinoembryonic antigen-related cell adhesion molecule 6 (CEACAM6), which is abnormally expressed in the enterocytes from CD patients [7]. In addition, AIEC exacerbate intestinal inflammation in CEABAC10 transgenic mice expressing human CEACAM6 [8]. These observations suggested that AIEC play an important role in CD etiopathogenesis.

In the past few years, genome-wide associations and functional studies have raised autophagy as a crucial pathway that is implicated in CD etiology [9]. Autophagy is a tightly regulated homeostatic process responsible for the elimination of damaged cytosolic components via the lysosomal pathway [9,10,11]. We have shown that upon AIEC infection, autophagy is induced in host cells to control the intracellular replication of the bacteria [12,13,14]. The CD-associated polymorphisms in genes involved in autophagy *ATG16L1* and *NOD2* lead to a defect in autophagy-mediated control of AIEC intracellular replication with a consequent increase in pro-inflammatory responses [13,15,16]. Furthermore, genetically modified mice exhibiting defective autophagy have increased intestinal colonization by AIEC and aggravated inflammation, compared to wild-type mice [12,17]. In addition, we have reported that AIEC can modulate the levels of several host microRNAs (miRNA, miR) to impair the autophagy response in IECs [14]. These observations suggested that autophagy is a key actor of CD physiopathology, and that AIEC can hijack this function via a post-transcriptional regulatory process in CD patients who do not carry autophagy-related risk variants.

SUMOylation was identified in 1997 as a reversible post-translational protein modification affecting a wide range of proteins within the cells [18]. SUMOs (small ubiquitin-related modifiers) are small peptides of ~10 kDa expressed throughout the eukaryotic kingdom. Four distinct SUMOs have been identified in the human genome: SUMO1, 2 and 3 are ubiquitously expressed, whereas SUMO4 is expressed only in the spleen, lymph nodes, and kidney. SUMOylation is the formation of an isopeptide bond between the carboxyl-terminal Gly residue of a SUMO and the Lys side chain of the acceptor protein. Most of the SUMOylation sites follow a canonical consensus motif of ψ-K-x-E (ψ is a hydrophobic amino acid, including A, I, L, M, P, F, or V, while x is any amino acid residue) [18]. The conjugation process requires three steps in which specialized enzymes are involved. First, SUMO protein is activated by an E1 enzyme, the SUMO-activating enzyme (SAE) 1/SAE2 heterodimer. Next, SUMO is transferred to ubiquitin conjugase 9 (UBC9), the unique E2 conjugating enzyme of the SUMOylation machinery. Finally, SUMO is transferred to the substrate, a process facilitated by E3 ligases named PIAS (protein inhibitors of activated STAT) [18]. In mammalian cells, four PIAS have been identified [19]. Once conjugated to its substrate, SUMO can be deconjugated by different SUMO isopeptidases called sentrin-specific proteases (SENP1-3 and SENP5-7), which tightly regulate the SUMOylation levels of proteins [18]. Whereas several viruses have been found to interfere with SUMOylation process [20], only few pathogenic bacteria have been reported to do so such as *Yersinia pestis* [21], *Listeria monocytogenes* [22], *Shigella flexneri* [23,24], *Salmonella* Typhimurium [25], *Anaplasma phagocytophilum* [26], *Ehrlichia chaffeensis* [27], colorectal cancer-associated *E. coli* [28] and the plant pathogen *Xanthomonas campestris* [29]. So far, a role for SUMOylation in CD-associated AIEC infection remains unknown.

In the current study, we investigated whether the SUMOylation of host IECs is modulated in response to AIEC infection and the potential involvement of this post-translational modification in AIEC pathogenesis.

## 2. Materials and Methods

### 2.1. Bacterial Strains

The AIEC LF82 reference strain isolated from a chronic ileal lesion of a CD patient [30], and the non-pathogenic *E. coli* MG1655 strain were used. The plasmid pFPV25.1, which harbors the green fluorescent protein (GFP), was used to visualize the LF82 bacteria by confocal microscopy. The AIEC strain LF82 was deleted for *fimH* (LF82Δ*fimH*), or deleted for type VI secretion systems (LF82Δ*T6SS*) have been previously generated [31,32]. Bacteria were grown in Luria–Bertani (LB) broth or on LB agar plates overnight at 37 °C.

### 2.2. Cell Culture

The intestinal epithelial T84 cell line (ATCC, CCL-248) was maintained in an atmosphere containing 5% CO_2_ at 37 °C in the culture medium recommended by ATCC (Molsheim, France).

### 2.3. Infection and Invasion Assay

Cells were seeded on 12- or 24-well plates and infected at a multiplicity of infection (MOI) of 10 bacteria per cell. Invasion assays were performed as previously described [14]. Briefly, after three hours of incubation in the culture medium without antibiotics, cells were washed with phosphate buffer saline (PBS), and the culture medium containing 100 μg/mL gentamicin was added for the indicated times. Cells were lysed with 1% Triton X-100 (Sigma, Saint-Quentin Fallavier, France) in deionized water. Samples were serially diluted and plated onto LB agar plates, and the number of bacteria was determined by counting the colony-forming units (CFU). When indicated, cells were pre-treated for four hours with 100 µM of anacardic acid (Sigma) [33], or 30 minutes with 0.5 µg/mL of colchicine (Sigma) [34].

### 2.4. Protein Extraction and Western Blot Analysis

Cells were lysed in radioimmune precipitation assay buffer (150 mM NaCl, 0.5% sodium deoxycholate, 50 mM Tris-HCl pH 8.0, 0.1% SDS, 0.1% Nonidet P-40) supplemented with protease inhibitors (Roche, Boulogne-Billancourt, France) and 5 mM *N*-ethylmaleimide (Sigma). Proteins were separated on SDS/PAGE gels, transferred to nitrocellulose membranes (Amersham Biosciences, Velizy-Villacoublay, France), blocked with 5% non-fat milk in PBS containing 0.1% Tween-20. Membranes were then probed overnight at 4°C with the relevant primary antibodies: anti-SUMO1 (Sigma), anti-SUMO2/3 (Sigma), anti-LC3 (Sigma), anti-SAE1 (Abcam, Paris, France), anti-SAE2 (Abcam), anti-UBC9 (Abcam), anti-PIAS3 (Cell Signaling Technology, Saint Quentin Yvelines, France), anti-p62 (Cell Signaling Technology), anti-β-actin (Cell Signaling Technology), and anti-GAPDH (Cell Signaling Technology). After washes, membranes were incubated with the appropriate HRP-conjugated secondary antibodies (Cell Signaling Technology), and blots were detected using the Enhanced Chemiluminescence Detection kit (Amersham Biosciences, Charfent, UK).

### 2.5. Quantitative RT-PCR (qRT-PCR)

Total RNAs were isolated using the TRIzol reagent (Invitrogen, Dardilly, France) or miRNeasy kit (Qiagen, Courtaboeuf, France) following the manufacturer’s instruction. One microgram of total RNAs was reverse-transcribed by using the NCode^TM^ miRNA first-strand cDNA synthesis kit (Invitrogen) to quantify mature microRNA (miRNA) levels, or using the first-strand cDNA synthesis kit (Euromedex) to quantify mRNA expression levels. Quantitative RT-PCR (qRT-PCR) (quantitative Reverse-Transcription Polymerase Chain Reaction) was performed on a Mastercycler Realplex^4^ (Eppendorf, Montesson, France) using SYBR Green qPCR Master Mix (Roche) and specific primers (Table S1). qRT-PCR analysis of miR-18 and U6 were performed using forward primers (Table S1) and a universal reverse primer provided by the NCode^TM^ miRNA first-strand cDNA synthesis kit. Human β-actin and mouse 36B4 were used as internal controls for quantification of mRNA. U6 was used as an internal control for quantification of miR-18 expression. The fold-induction was calculated by using the *Ct* method as follows: ΔΔ*Ct* = (*Ct*_target gene_ − *Ct*_internal control_)_treatment_ − (*Ct*_target gene_ − *Ct*_internal control_)_nontreatment_, and the final data were derived from 2^−ΔΔ*Ct*^.

### 2.6. Enzyme-Linked Immunosorbent Assays (ELISA)

The amount of IL-8 secreted in the cell culture supernatants was determined by ELISA (R&D systems, Minneapolis, MN, USA) according to the manufacturer’s instructions.

### 2.7. Transfection Experiments

Cells cultured on 12-well plastic plates were transfected with 500 ng of plasmid or 30 nM of miRNA precursor or anti-miRNA (Ambion, Austin, TX, USA) using Lipofectamine 2000 (Invitrogen) and OPTI-MEM-reduced serum medium (Invitrogen), according to the manufacturer’s instructions. Cells were changed to fresh growth medium after 8 h, and infected 24 h later.

### 2.8. Generation of the T84 Cell Line containing a UBC9-shRNA Construct

2 × 10^5^ T84 cells seeded on a 6-well plate were transfected with the *UBC9* short hairpin RNA (shRNA) plasmid or an empty vector (Santa Cruz, Heidelberg, Germany), as described above. Cells were stably selected in DMEM/F12 medium containing 10 µg/mL puromycin (Sigma).

### 2.9. Fluorescent Microscopy

Cells seeded on coverslips were infected with the LF82-GFP strain at a MOI of 10 for 3 h, washed with PBS, and the culture medium containing 100 μg/mL gentamicin was added for 12 h. Cells were then fixed with 4% paraformaldehyde, permeabilized with 0.5% Triton X-100 for 20 min, blocked with PBS containing 0.025% Triton X-100, 3% bovine serum albumin (BSA), and 5% fetal bovine serum (FBS). The actin cytoskeleton was stained for 15 min using tetramethylrhodamine (TRITC)–phalloidin (Sigma). Nuclei were stained with Hoechst 33342 (Sigma). Coverslips were then mounted with a Mowiol solution (Calbiochem, San Diego, CA, USA). Slides were examined with a Zeiss LSM 510 Meta confocal microscope. Each confocal microscopy image is representative of three independent experiments.

### 2.10. In Vivo Infection

Carcinoembryonic antigen bacterial artificial chromosome (CEABAC) 10 transgenic mice were infected as previously described [8]. Mice were sacrificed 24 h after infection.

### 2.11. Isolation of Intestinal Epithelial Cells (IECs)

IECs were isolated as previously described [35]. Briefly, mouse intestines were dissected and flushed with a solution containing 154 mM NaCl and 1 mM Dithiothreitol (DTT), to remove fecal contents. The intestinal segments were ligated, filled with PBS, and incubated in PBS at 37 °C. After 15 min, PBS was substituted with PBS supplemented with 1.5 mM Ethylenediaminetetraacetic acid (EDTA) and 0.5 mM DTT. After 30 min at 37 °C, one ligature was removed, and the contents were collected. The recovered IECs were washed twice in PBS by centrifugation at 1300 rpm for 5 min, and they were used for the preparation of protein extracts.

### 2.12. Ethics Statement

This study was carried out in strict accordance with the recommendations of the Guide for the Care and Use of Laboratory Animals of the University of Clermont Auvergne (Clermont-Ferrand, France). The animal protocol was approved by the Committee for Research and Ethical Issues of the Department of Auvergne (CEMEA Auvergne; Permit Number: CEMEAA, 2015032716314007) following international directive 86/609/CEE (n°CE16-09). Informed written consent was obtained from all patients to isolate *E. coli* strains from biopsies or stools (CCPPRB Lille 1994 number 94/01). All samples were anonymized.

### 2.13. Statistical Analysis

Values were expressed as means ± SEM. Statistical analyses between two or several groups were performed using Student’s t-test (Mann–Whitney if not parametric) or ANOVA followed by a post-test Bonferroni (Kruskal–Wallis if not parametric) with GraphPad Prism version 6 software. A *p* value of less than 0.05 was considered to be statistically significant. * *p* < 0.05; ** *p* ≤ 0.005; *** *p* ≤ 0.001.

## 3. Results

### 3.1. AIEC Decreases SUMO-Conjugated Proteins in IECs

**Figure 1:**
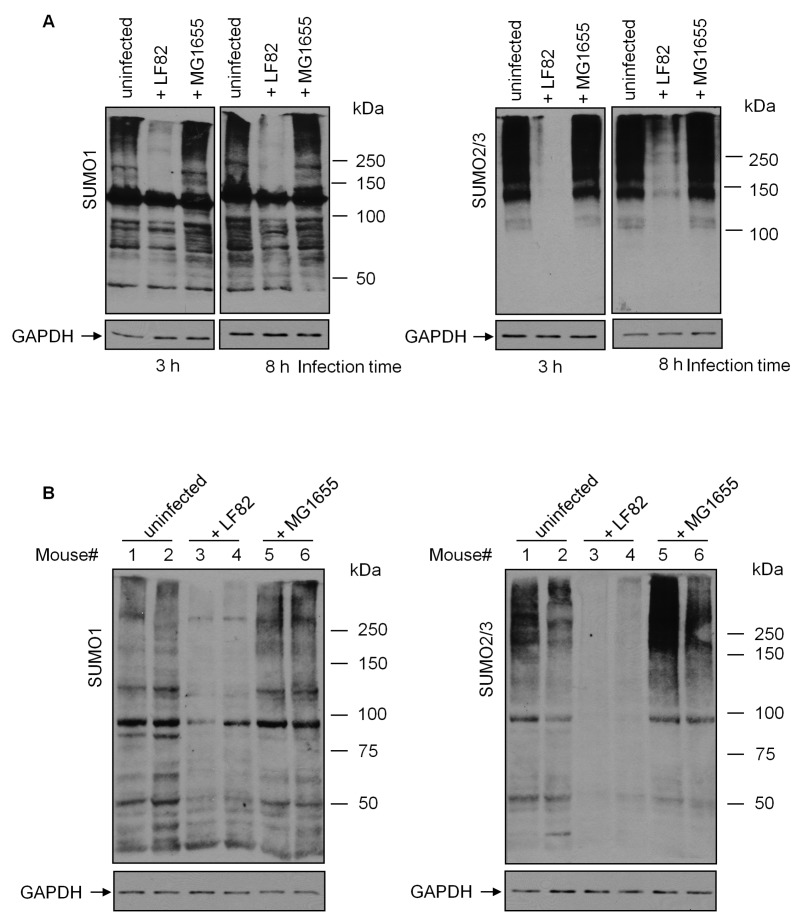
AIEC infection decreases SUMO-conjugated protein levels in intestinal epithelial T84 cells. (**A**) T84 cells were infected with the AIEC LF82 or the non-pathogenic *E. coli* MG1655 strain, and the SUMO-conjugated protein profiles were analyzed by Western blot. (**B**) CEABAC10 transgenic mice were infected with the LF82 or the MG1655 strain by gavage. After infection, the mice were sacrificed, and the ileal epithelial cells were isolated, as described in the Materials and Methods. SUMO-conjugated protein profiles of mouse enterocytes were analyzed by Western blot.

To investigate whether AIEC are able to modify the SUMOylation of host cells, we compared the global pattern of SUMO1- or SUMO2/3-conjugated proteins in human intestinal epithelial T84 cells uninfected or infected with the AIEC LF82 reference strain. We found that three hours of L82 infection, unlike an infection with the non-pathogenic *E. coli* K12 MG1655 strain, induced a profound decrease in both SUMO1- and SUMO2/3-conjugated protein levels, compared with uninfected cells (Figure 1A). In addition, AIEC-induced protein deSUMOylation persisted until eight hours post-infection (Figure 1A). In an effort to confirm the modification of host protein SUMOylation by AIEC infection, CEABAC10 transgenic mice expressing human CEACAM6, the host receptor required for AIEC adherence [7], were infected with LF82 or MG1655 bacteria by gavage, as previously described [8]. In consistence with the in vitro data, a marked decrease in SUMO1- and SUMO2/3-conjugated protein levels was observed in IECs isolated from LF82-infected CEABAC10 transgenic mice, compared to uninfected mice (Figure 1B). Infection of CEABAC10 transgenic mice with the MG1655 strain; however, did not exhibit any significant effect on SUMO-conjugated protein profiles in IECs (Figure 1B). Together, these results demonstrate that AIEC are able to induce a massive protein deSUMOylation in IECs.

### 3.2. SUMOylation in IECs Affects AIEC Intracellular Replication and Inflammatory Response

**Figure 2:**
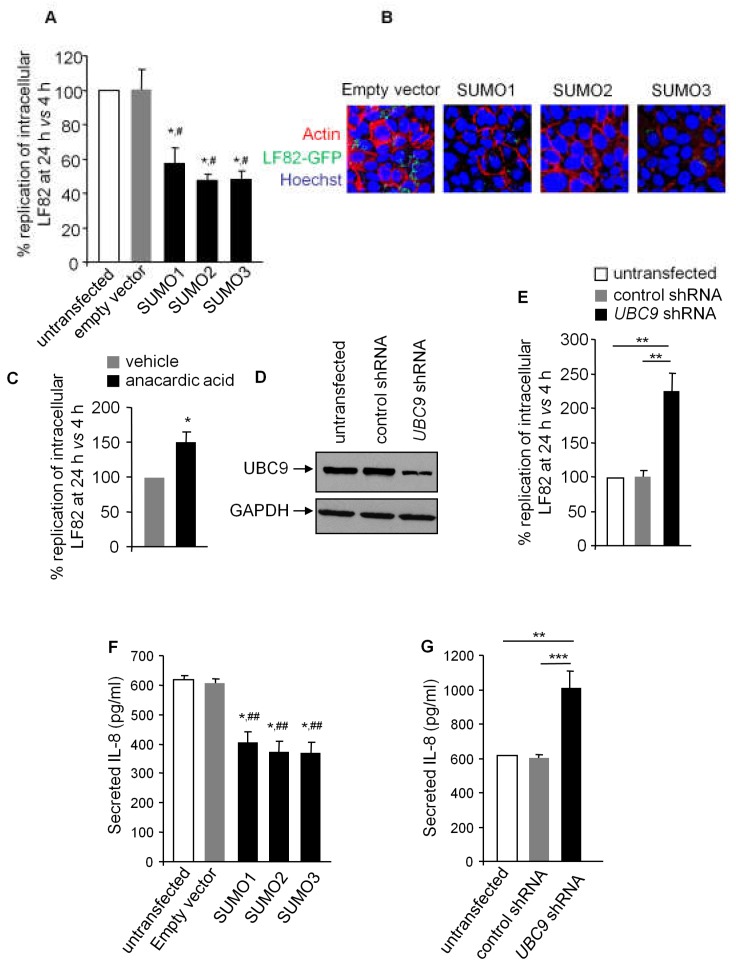
SUMOylation controls AIEC intracellular replication and AIEC-induced inflammation. (**A**,**B**,**F**) T84 cells were transfected with SUMOs-encoding plasmids before being infected with LF82 (**A**) or LF82-GFP (**B**). (**A**) Intracellular LF82 number was counted on LB agar plates and is presented as the ratio of intracellular bacteria at 24 h vs. 4 h post-infection, compared to untransfected condition, considered as 100%. (**B**) Representative confocal micrographs of cells infected with LF82-GFP (green) and immunolabeled for actin (red). Nuclei were stained with Hoechst (blue). (**C**) T84 cells pretreated with anacardic acid or vehicle were infected with the LF82 strain. LF82 intracellular number was counted on LB agar plates and is presented as the ratio of intracellular bacteria at 24 h vs. 4 h post-infection, compared to vehicle, considered as 100%. (**D**,**E**,**G**) T84 cells were stably transfected with *UBC9* shRNA or an empty shRNA. (**D**) UBC9 expression level was analyzed by Western blot. (**E**) Untransfected or transfected cells were infected with the LF82 strain. LF82 intracellular number was counted on LB agar plates and is presented as the ratio of intracellular bacteria at 24 h vs. 4 h post-infection, compared to untransfected condition, considered as 100%. (**F**,**G**) T84 cells were infected with the LF82 strain. Secreted IL-8 amounts in cell culture supernatant were quantified by ELISA, 24 h post-infection. Data are means ± SEM of three replicates and are representative of three independent experiments. (**A**,**F**) (* *p* < 0.05; ** *p* ≤ 0.005) vs. untrasfected; (^#^ *p* < 0.05; ^##^ *p* ≤ 0.005) vs. empty vector. (**C**,**E**,**G**) * *p* < 0.05; ** *p* ≤ 0.005; *** *p* ≤ 0.001.

We next investigated the functional consequences of AIEC-induced protein deSUMOylation in IECs. For this purpose, T84 cells transiently transfected with an empty vector, or a vector encoding SUMO1, SUMO2, or SUMO3, were infected with LF82. We found that over-expression of SUMOs significantly reduced LF82 intracellular replication, determined by gentamicin protection assay, compared to cells transfected with an empty vector (Figure 2A). Confocal microscopic analysis using a LF82-GFP strain consistently showed decreased numbers of intracellular LF82 in SUMO-overexpressing cells, compared to empty vector-transfected cells (Figure 2B). In order to confirm the importance of protein SUMOylation in the control of LF82 intracellular replication, T84 cells were pre-treated with anacardic acid, an inhibitor of the SUMOylation process [33], and infected with the LF82 strain. Anacardic acid treatment significantly increased LF82 intracellular replication (Figure 2C) without modifying cellular viability (data not shown). To strengthen these results, we generated a T84 cell line in which UBC9 expression was depleted. For this, cells were stably transfected with a shRNA directed against *UBC9* (*UBC9* shRNA) or a control shRNA. As shown in Figure 2D, UBC9 expression was decreased in *UBC9* shRNA-transfected cells, compared to untransfected or control shRNA-transfected cells. Interestingly, LF82 intracellular replication was significantly higher in cells with depleted UBC9 expression (Figure 2E). Since AIEC have been shown to induce a pro-inflammatory response in host IECs [3], we assessed whether over-expression of SUMOs or depletion in UBC9 expression could modify the production of the pro-inflammatory chemokine IL-8 upon LF82 infection. As shown in Figure 2F, IL-8 production was significantly lower in SUMO-transfected T84 cells compared to untransfected and empty vector-transfected cells. Furthermore, IL-8 production was increased in *UBC9* shRNA-transfected cells compared to untransfected or control shRNA-transfected cells (Figure 2G). These results show that SUMOylation has a role in restricting AIEC intracellular replication, and inhibiting pro-inflammatory response. Consequently, SUMOylation hijacking mediated by LF82 may favor its replication, and it contributes to its pro-inflammatory activity in CD patients.

### 3.3. SUMOylation Regulates the Autophagy Response

**Figure 3:**
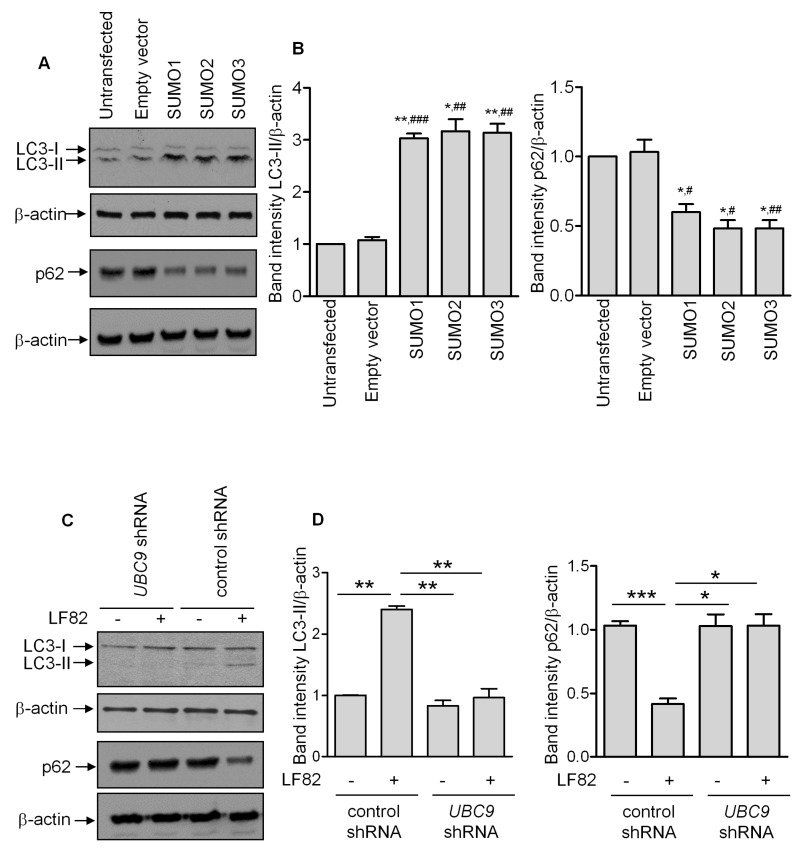
SUMOylation regulates autophagy response. (**A**) Western blot analysis for the shift of LC3-I toward LC3-II and p62 levels in T84 cells transfected with SUMO-encoding plasmids or an empty vector. (**B**) Quantification of LC3-II/β-actin or p62/β-actin band intensity from three independent blots. (* *p* < 0.05; ** *p* ≤ 0.005) vs. untrasfected; (^#^ *p* < 0.05; ^##^ *p* ≤ 0.005; ^###^ *p* ≤ 0.001) vs. empty vector. (**C**) T84 cells stably transfected with *UBC9* shRNA or an empty shRNA were infected with the LF82 strain. The shift of LC3-I toward LC3-II and p62 levels were analyzed by Western blot. (**D**) Quantification of LC3-II/β-actin or p62/β-actin band intensity from three independent blots. * *p* < 0.05; ** *p* ≤ 0.005; *** *p* ≤ 0.001.

As we showed the importance of SUMOylation in the control of LF82 intracellular proliferation, which is tightly regulated by autophagy [12,13,14,15], we next investigated whether protein SUMOylation is involved in the regulation of the autophagic process. As shown in Figure 3A,B, over-expression of SUMO1, SUMO2, or SUMO3 in T84 cells induced autophagy, as characterized by a shift from LC3-I (the free cytosolic form) toward LC3-II (the autophagosomal form) [11], compared with untransfected cells or cells that are transfected with an empty vector. Induction of a functional and degradative autophagy flux in SUMOs-transfected T84 cells was confirmed by decreased levels of p62 (Figure 3A,B), a receptor protein incorporated into the autophagosome and degraded inside autolysosomes [11].

In addition, induction of autophagy in response to LF82 infection was suppressed when SUMOylation was inhibited due to *UBC9* depletion (Figure 3C,D). Together, our results suggest that deSUMOylation induced by AIEC infection might inhibit autophagy response, allowing the bacteria to replicate intracellularly and to trigger a strong pro-inflammatory response.

### 3.4. Adhesion of LF82 is Required for the Induction of deSUMOylation

**Figure 4:**
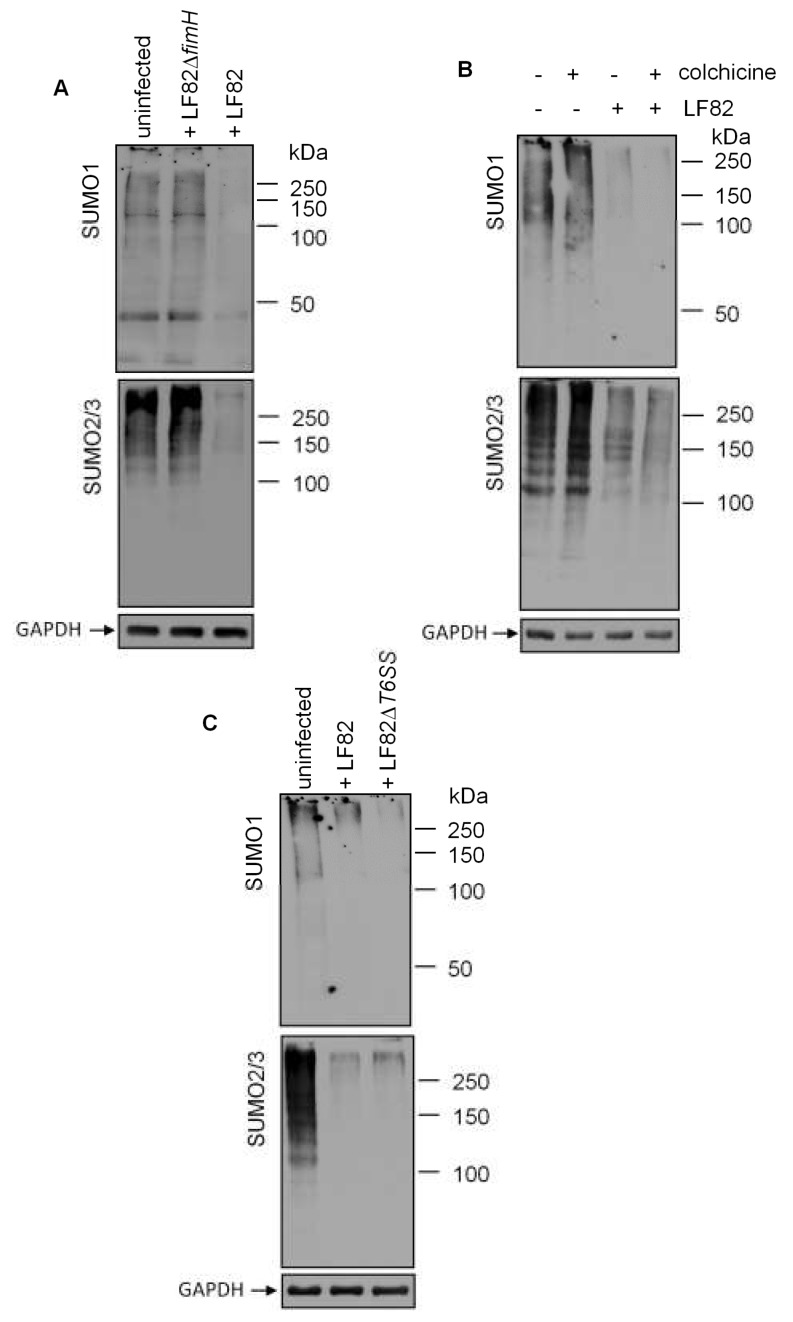
Adhesion of LF82 is required for deSUMOylation. (**A**,**C**) T84 cells were infected with wild-type AIEC LF82, LF82Δ*fimH*, or LF82Δ*T6SS*, and the SUMO-conjugated protein profile was analyzed by Western blot. (**B**) T84 cells were pre-treated with colchicine at 0.5 µg/mL for 30 min, and then infected with AIEC LF82. The SUMO-conjugated protein profile was analyzed by Western blot.

We next investigated the mechanisms by which LF82 bacteria impaired SUMOylation. It is well-established that the pathogenesis of AIEC requires bacterial adhesion via FimH [3,36], a protein expressed at the top of the type 1 pili. As shown in Figure 4A, loss of FimH (LF82Δ*fimH*) abrogated LF82-induced deSUMOylation, demonstrating that LF82 adhesion is necessary for the deSUMOylation process. Another important feature of AIEC pathogenesis is their ability to survive and to grow inside eukaryotic cells [3]. Interestingly, the use of colchicine, a potent inhibitor of LF82 cellular invasion [34], did not prevent LF82-induced deSUMOylation (Figure 4B), demonstrating that AIEC-induced deSUMOylation does not require the host cell invasion by bacteria. Altogether, our results suggest that LF82 adhesion is a critical step for inducing host protein deSUMOylation.

To date, only three bacterial effectors have been shown to be involved in deSUMOyaltion: *Listeria* listeriolysin O [22], *Xanthomonas* XopD [29] and *Yersinia* YopJ [21]. Genome sequencing of the LF82 [37] did not reveal any homologous proteins or proteins harboring SUMO protease domain. However, XopD and YopJ are secreted inside the host via a type III secretion system. Even if LF82 does not possess any type III secretion system, these bacteria harbor two pathogenicity islands of 30.5 kb and 35 kb that encode for two type VI secretion systems (T6SS) [37]. Despite their recent discovery in 2010, the number of characterized T6SS effector proteins is rapidly growing. It was tempting to speculate that bacterial T6SS secrete proteins that might impact SUMOylation of host cells. However, deletion of the two T6SS-encoding pathogenicity islands (LF82Δ*T6SS*) [32] did not impair LF82-induced deSUMOylation (Figure 4C). Altogether, our data demonstrate that LF82 adhesion is required for the induction of deSUMOylation in IECs.

### 3.5. PIAS3 Downregulation is Associated with SUMOylation Hijacking Mediated by LF82 in IECs

**Figure 5:**
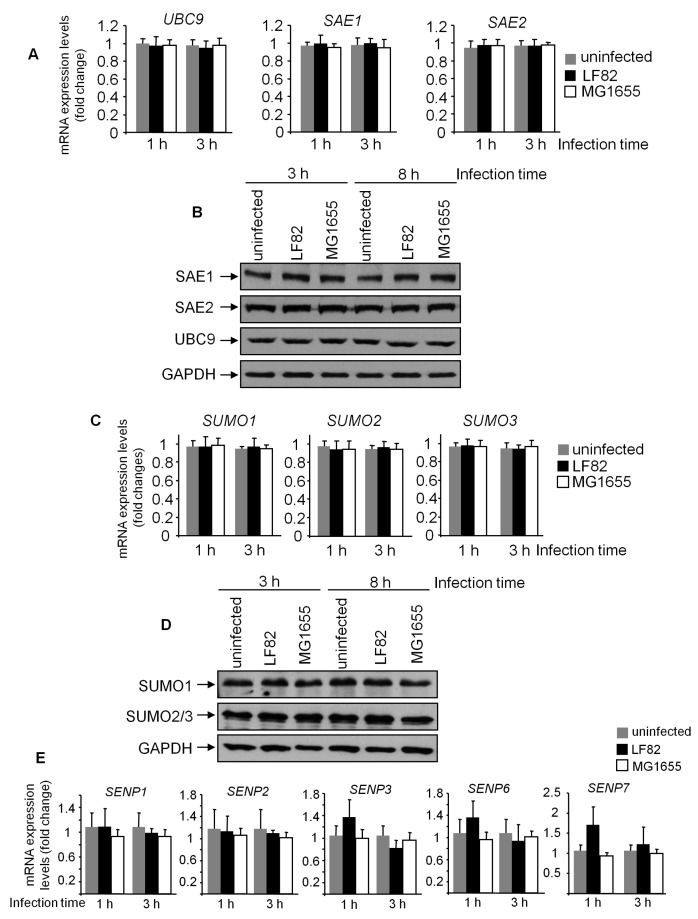
AIEC infection does not modify expression levels of SAEs, UBC9, SUMOs and SENPs. T84 cells were infected with the AIEC LF82 or the non-pathogenic *E. coli* MG1655 strain for the indicated time. mRNA expression levels of *SAE1*, *SAE2*, *UBC9* (**A**), *SUMO*s, (**C**) as wells as *SENP*s (**E**) were analyzed by qRT-PCR. (**B**,**D**) Protein levels of SAE1, SAE2, UBC9, (**B**) as well as SUMOs, (**D**) were analyzed by Western blot. Data are means ± SEM of three replicates, and representatives of three independent experiments.

**Figure 6:**
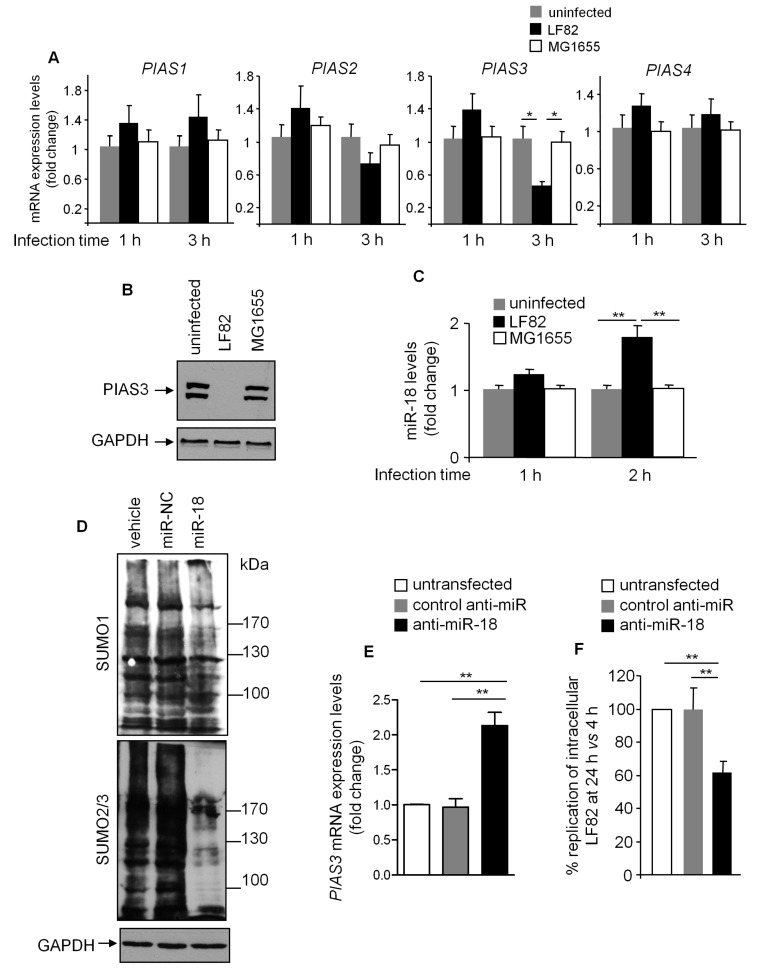
MiR-18 is involved in AIEC-induced deSUMOylation in T84 cells. T84 cells were infected with the AIEC LF82 or the non-pathogenic *E. coli* MG1655 strain for the indicated time. (**A**) *PIAS* mRNA expression levels were assessed by qRT-PCR. (**B**) T84 cells were infected for 3 h, and PIAS3 protein level was assessed by Western blot. (**C**) MiR-18 levels were analyzed by qRT-PCR. (**D**) T84 cells were transfected with a precursor of miR-18 or a miRNA-negative control (miR-NC), and SUMO-conjugated protein profile was analyzed by Western blot. (**E**,**F**) Untransfected T84 cells or cells transfected with anti-miR-18 or a control anti-miR were infected with the LF82 strain. (**E**) *PIAS3* mRNA expression level was assessed by qRT-PCR. (**F**) LF82 intracellular number was counted on LB agar plates and is presented as the ratio of intracellular bacteria at 24 h vs. 4 h post-infection, compared to untransfected condition, considered as 100%. Data are means ± SEM of three replicates, and they are representative of three independent experiments. * *p* < 0.05; ** *p* ≤ 0.005; *** *p* ≤ 0.001.

SUMOylation can be affected by the expression level of SUMO peptides and is under the control of several enzymes, including SAE1, SAE2, and UBC9, which are in charge of SUMO activation and their transfer to the substrate [18]. To investigate the mechanism underlying the AIEC-induced protein deSUMOylation in T84 cells, we analyzed expression levels of UBC9, SAE1, and SAE2 by qRT-PCR and Western blot. Figure 5A,B show that LF82 infection did not modify the expression of these enzymes at both the mRNA and protein levels. We next investigated whether LF82 infection induces a decrease in total SUMO1 and SUMO2/3 expression. As shown by qRT-PCR and Western blot analyses, LF82 infection did not modify the expression of SUMOs (Figure 5C,D). We then hypothesized that the AIEC-induced decrease in protein SUMOylation might be due to a modification of expression of SENPs, which are involved in SUMO deconjugation required for a tight regulation of protein SUMOylation levels [18]. However, as shown in Figure 5E, LF82 infection did not significantly modify mRNA expression levels of different *SENPs*. We finally investigated expression levels of the E3 ligases PIAS, which are involved in the transfer of SUMOs to their substrates [18]. While mRNA expression levels of *PIAS1*, *2*, and *4* in T84 cells were not changed upon LF82 infection, *PIAS3* expression was significantly decreased (Figure 6A). More importantly, Western blot analysis showed that infection of T84 cells with the LF82 strain, but not with the MG1655 strain, decreased PIAS3 protein expression (Figure 6B). These results suggest that LF82 decreases protein SUMOylation by targeting PIAS3 expression.

### 3.6. LF82 Downregulates PIAS3 Expression via Upregulating miR-18 Level

It was reported that PIAS3 expression is negatively controlled by miR-18 [38]. Interestingly, LF82 infection induced an increase in miR-18 level as early as two hours post-infection (Figure 6C). Interestingly, when T84 cells were transfected with a precursor of miR-18, we observed a decrease in SUMO1- and SUMO2/3-conjugated protein levels with a more profound effect for SUMO2/3-conjugated proteins, as observed after LF82 infection (Figure 6D). This was not observed in cells transfected with a miR-negative control (Figure 6D). These results suggest that AIEC may inhibit protein SUMOylation in IECs by increasing miR-18 levels to suppress PIAS3 expression.

Since we identified miR-18 as a potential regulator of SUMOylation upon LF82 infection, we transfected T84 cells with an antisense of miR-18 (anti-miR-18), infected them with LF82 and determined the number of intracellular bacteria. As shown in Figure 6E, transfection with anti-miR-18, but not a negative control anti-miRNA, increased *PIAS3* mRNA expression level in LF82 infected cells. We found that the number of intracellular LF82 was decreased in cells transfected with anti-miR-18, compared to untransfected cells or cells that are transfected with negative-control anti-miRNAs (Figure 6F). Together, these results demonstrate the role of miR-18-regulated SUMOylation in the control of AIEC intracellular replication.

## 4. Discussion

Post-translational modifications are importantly involved in the complex crosstalk between pathogens and their host cells. While viruses have been known to modulate SUMOylation over many years [20], the impact of bacteria on this post-translational modification remains largely unknown. In the current study, we found that AIEC infection decreases the global protein SUMOylation pattern in IECs, in vitro, as well as in vivo.

Except for *A. phagocytophilum* [26], protein SUMOylation appears to be required to control bacterial intracellular survival. This has been reported for *S. flexneri* [23], *L. monocytogenes* [22], and *S.* Typhimurium [25]. Similarly, we showed in the current study that the AIEC-induced decrease of protein SUMOylation directly impacts AIEC intracellular survival. Indeed, the over-expression of SUMOs limits the replication of AIEC in human intestinal epithelial T84 cells. Furthermore, the depletion of UBC9 or treatment with anacardic acid, an inhibitor of SUMOylation, significantly increases the proliferation of AIEC inside T84 cells. We thus sought to investigate the mechanism underlying the regulation of AIEC intracellular replication by SUMOylation. To date, there are only a few reports showing the role of proteins that are regulated by SUMOylation in host response to pathogen infection. It has been shown that SUMOylation of SMAD4 is necessary to respond to TGF-β which is involved in host resistance against infection with *L. monocytogenes* [22]. The plant pathogen *X. euvesicatoria* was shown to deSUMOylate the transcription factor SIERF4 repressing ethylene production required for anti-*X. euvesicatoria* immunity [39]. Finally, *Shigella* modifies SUMO-conjugated transcriptional regulators that are involved in intestinal functions and inflammatory responses [23]. In the case of AIEC infection, it has been shown that autophagy is a crucial host defense mechanism for controlling the intracellular replication of the bacteria [12,13,14,15]. Thus, we investigated the impact of SUMOylation on the autophagy response to AIEC infection. Interestingly, we observed that over-expression of SUMOs induces autophagy, and it inhibits AIEC intracellular replication. In addition, the inhibition of SUMOylation suppresses the autophagy response to AIEC infection, and it increases AIEC intracellular replication. These results suggest that AIEC induces deSUMOylation to impair the autophagy defense of the host, favoring their intracellular replication.

The mechanisms by which pathogenic bacteria modulate host protein SUMOylation have only recently started to be explored, and it remains poorly understood, to date. Two main mechanisms have been identified: bacteria induce a decrease in expression of the enzymes that are involved in SUMOylation, or a bacterial protein after being injected into host cell is able to impair SUMOylation. *Listeria monocytogenes*, *Shigella flexneri* and *Salmonella* Typhimurium have been reported to induce a massive decrease of protein SUMOylation in human cells, and this is due to the inhibition of UBC9 expression [22,24,25]. It should be noted that in the case of *L. monocytogenes*, the secreted toxin listeriolysin O is responsible for the down-regulation of UBC9, while for *S.* Typhimurium, the bacterial effector remains unknown. In the case of AIEC, we showed that expression levels of UBC9 as well as SAE1 and SAE2 enzymes were not significantly modified upon infection. However, expression of the SUMO E3 ligase PIAS3 was decreased upon AIEC infection. This decrease in PIAS3 expression might explain, at least in part, a decrease in SUMO-conjugated proteins. It is known that miR-18 negatively regulates PIAS3 expression [38]. Interestingly, miR-18 is up-regulated upon AIEC infection, and the use of an anti-miR-18 significantly decreased host SUMOylation, and consequently impaired AIEC intracellular proliferation.

It has been shown that *Xanthomonas* XopD and *Yersinia* YopJ effectors (two bacterial proteins injected inside the host) mimic endogenous SUMO isopeptidase of the host, thereby inducing a massive deSUMOylation [21,29]. Based on the sequence of AIEC LF82 genome [37], no homologous proteins, as well as no protein harboring a SUMO protease domain were predicted to be expressed in AIEC LF82. However, two T6SS have been identified [37]. Here, we observed that the deletion of those T6SS did not impair LF82-induced deSUMOylation, suggesting that bacterial effectors secreted by T6SS are not involved in the deSUMOylation process. One feature of AIEC is their ability to persist inside the cell [3]. Thus, we hypothesized that intracellular bacteria might induce deSUMOylation. However, we observed that the inhibition of bacterial invasion by the use of colchicine did not block LF82-induced deSUMOylation. Colchicine inhibits bacterial invasion, but it does not block bacterial adhesion, suggesting that adhesion might be the critical step in the deSUMOylation process. In addition, that LF82Δ*fimH* mutant was unable to adhere to host cells did not modify global protein SUMOylation. Altogether, our data suggested that AIEC bacterial attachment to host cells is sufficient, and necessary to induce deSUMOylation in infected cells.

In conclusion, the present study suggests that upon adhesion to host cells, CD-associated AIEC impairs host autophagy, to replicate intracellularly by modulating protein SUMOylation partly via miR-18-mediated inhibition of PIAS3 expression.

## Supplementary Materials

The following are available online at http://www.mdpi.com/2073-4409/8/1/35/s1, Table S1: Primers used in this study.
