# Supplementary material for: Crohn’s Disease-Associated Adherent-Invasive Escherichia coli Manipulate Host Autophagy by Impairing SUMOylation
Source: Cells. 2019 Jan 9;8(1):35. doi: 10.3390/cells8010035 (PMC6357120; doi:10.3390/cells8010035)
Supplement: Supplementary file 1 [file cells-08-00035-s001.pdf]

Supplementary Table 1: Primers used for qRT-PCR.

Primer	Sequence (5'-3')
β-actin$_{For}$	GTC ACC CAC ACT GTG CCC ATC
β-actin$_{Rev}$	ACG GAG TAC TTG CGC TCA GGA
IL-8$_{For}$	GTG CAG TTT TGC CAA GGA GT
IL-8$_{Rev}$	AAA TTT GGG GTG GAA AGG TT
SUMO1$_{For}$	GGA TTT GTA AAC CCC GGA GC
SUMO1$_{Rev}$	TCC CCC AAG TCC TCA GTT GAA
SUMO2$_{For}$	AGA TTC CGA TTT GAC GGG CA
SUMO2$_{Rev}$	CAG TAG ACA CCT CCC GTC TG
SUMO3$_{For}$	GAG AGG CAG GGC TTG TCA AT
SUMO3$_{Rev}$	GCT GGA ACA CGT CGA TGG
Ubc9$_{For}$	CCT CAG CAG ACT CGC CCA GGA
Ubc9$_{Rev}$	GGC GCA CTC CCA GTT CAT GAG G
SAE1$_{For}$	GAG GCT GGC GGC GGC ATT AG
SAE1$_{Rev}$	CTC CAG TCC CCA CAG GCG GA
SAE2$_{For}$	GCA GCC GGC TTC AAG CAG ATG A
SAE2$_{Rev}$	GGC CCC ACT TTT TCC GGG GC
PIAS1$_{For}$	GCG GAC AGT GCG GAA CTA A
PIAS1$_{Rev}$	GTT TGC GTC CGT GCT TGT TT
PIAS2$_{For}$	TGG CGG ATT TCG AAG AGT TGA
PIAS2$_{Rev}$	TAA ATG CAG CGC CCT CAT CA
PIAS3$_{For}$	CGC CTG CGA TGT CTC AAG AT
PIAS3$_{Rev}$	GTG CTT CCG TCC ACT CTT GT
PIAS4$_{For}$	TGG TGA AGC TGC CGT TCT TT
PIAS4$_{Rev}$	CGT CAA TGC GAA GAT GCA CG
SENP1$_{For}$	GGT TCC CGG GTT TTG CGT
SENP1$_{Rev}$	CCA GCA TCC ATC CTC ATC CTA T
SENP2$_{For}$	TCT TGT GAA CTG ACA GGT TCT GG
SENP2$_{Rev}$	CCA AAG GAA GGC AGG ACT CT
SENP3$_{For}$	TGA TGG GGT GAA AAG GTG GAC
SENP3$_{Rev}$	CGC TGC GAG TCA AAA TAG GTG
SENP5$_{For}$	CTT CCC TTG GAG CTG ACG AA
SENP5$_{Rev}$	CAG CTG CTA CCC TCG TTC C
SENP6$_{For}$	CCA AAG CAG TCT GGA CCG AA
SENP6$_{Rev}$	ACC TTG GAG CCG ACT TAA CC
SENP7$_{For}$	CGG CCA TCT TCA TCC GAA ATC
SENP7$_{Rev}$	AGT CCA GCG TTC TGA GCT TC
miR-18$_{For}$	TAA GGT GCA TCT AGT GCA GAT
U6$_{For}$	CGC AAG GAT GAC ACG CAA ATT

For, forward; Rev, reverse.
